# Psychopharmakotherapie in Zeiten der COVID-19-Pandemie

**DOI:** 10.1007/s00115-020-00939-4

**Published:** 2020-06-02

**Authors:** J. Seifert, J. Heck, G. Eckermann, M. Singer, S. Bleich, R. Grohmann, S. Toto

**Affiliations:** 1 Klinik für Psychiatrie, Sozialpsychiatrie und Psychotherapie, Medizinische Hochschule Hannover, Carl-Neuberg-Straße 1, 30625 Hannover, Deutschland; 2 Institut für Klinische Pharmakologie, Medizinische Hochschule Hannover, Hannover, Deutschland; 3 Klinik für Forensische Psychiatrie und Psychotherapie, Bezirkskrankenhaus Kaufbeuren, Kaufbeuren, Deutschland; 4 Fachklinik für Psychiatrie, Psychotherapie und Psychosomatik, kbo-Lech-Mangfall-Klinik Agatharied, Hausham, Deutschland; 5 Klinik für Psychiatrie und Psychotherapie, Ludwig-Maximilians-Universität München, München, Deutschland

**Keywords:** Severe acute respiratory syndrome coronavirus 2 (SARS-CoV-2), Arzneimitteltherapiesicherheit, Infektion, Unerwünschte Arzneimittelwirkungen (UAW), Psychiatrie, Severe acute respiratory syndrome coronavirus 2 (SARS-CoV-2), Drug safety, Infection, Adverse drug reactions (ADR), Psychiatry

## Abstract

Im Rahmen der aktuellen *coronavirus disease 2019*(COVID-19)-Pandemie müssen sich viele Bereiche der Medizin umstrukturieren. Dies betrifft auch die Versorgung von Patienten mit psychischen Erkrankungen. Die Therapie psychischer Erkrankungen umfasst psychotherapeutische und psychopharmakologische Interventionen. Letztere können mit einer Vielzahl an unerwünschten Arzneimittelwirkungen (UAW) assoziiert sein, stellen aber in der aktuellen Situation mit Kontakt- und Ausgangsbeschränkungen die präferierte Therapieoption dar. Da der direkte Patientenkontakt zugunsten des Telefonats oder der Videokonferenz reduziert ist, müssen angepasste diagnostische und therapeutische Optionen gefunden werden, um eine ausreichende Patientensicherheit zu gewährleisten. Bedeutend sind hierbei die ausführliche Aufklärung der Patienten sowie eine aktive Abfrage von Symptomen zur rechtzeitigen Erkennung von UAW. Unter der Behandlung mit Psychopharmaka sind UAW zu befürchten, die besonders ungünstig sind, wenn sie im Rahmen einer akuten Infektion auftreten oder ein erhöhtes Infektionsrisiko begünstigen. Hierzu gehören Atemdepression, Agranulozytose, Intoxikation durch Hemmung des Arzneistoffmetabolismus und venöse Thromboembolien, die jeweils mit potenziell lebensbedrohlichen Folgen einhergehen. Gleichzeitig sollte auf eine ausreichende Wirksamkeit der Medikation geachtet werden, da die gegenwärtige Krise zu einer Exazerbation vorbestehender psychischer Erkrankungen führen bzw. deren Erstmanifestation begünstigen kann.

## Abstract (en)

In view of the current coronavirus disease 2019 (COVID-19) pandemic, patient care, including that of psychiatric patients, is facing unprecedented challenges. Treatment strategies for mental illness include psychotherapy and psychopharmacological interventions. The latter are associated with a multitude of adverse drug reactions (ADR); however, they may currently represent the preferred treatment due to restrictions regarding patient care (i.e. social distancing). Direct contact to patients may have to be reduced in favor of telephone calls or video conferences, so that new techniques in diagnosing and treating patients have to be established to guarantee patient safety. Patients should be extensively informed about relevant ADRs and physicians should actively ask patients about the timely recognition of ADRs. The use of psychotropic drugs may lead to an increased risk of developing ADRs, which are considered to be particularly unfavorable if they occur simultaneously with an acute infection or may even lead to an increased risk of infection. These include respiratory depression, agranulocytosis, intoxication by inhibition of metabolizing enzymes and venous thromboembolism, each of which may be associated with potentially fatal consequences; however, physicians should simultaneously ensure adequate efficacy of treatment, since the ongoing crisis may lead to a worsening of preexisting mental illnesses and to a surge in first onset of psychiatric disorders.

Seit Mitte März 2020 steht das Gesundheitssystem durch die *coronavirus disease 2019*(COVID-19)-Pandemie vor ungekannten Herausforderungen. Die Versorgungsstrukturen der Kliniken sind innerhalb kürzester Zeit darauf ausgelegt worden, die Behandlung von COVID-19-Erkrankten sowie die Abklärung von *severe acute respiratory syndrome coronavirus 2*(SARS-CoV-2)-Verdachtsfällen bestmöglich zu gewährleisten. Nichtsdestotrotz ist es essenziell, die Versorgung anderer Patienten nicht außer Acht zu lassen. Patienten mit psychischen Erkrankungen stellen ein besonders vulnerables Patientenkollektiv dar, das auch und vor allem in Zeiten der Pandemie unserer Aufmerksamkeit bedarf.

Viele Patienten mit psychischen Erkrankungen sind von funktionierenden Strukturen der Patientenversorgung abhängig, die aufgrund der aktuellen Hygienemaßnahmen nicht mehr uneingeschränkt verfügbar sind. Ambulante Behandler empfangen teilweise ihre Patienten nicht mehr persönlich, sondern nehmen den Kontakt telefonisch oder per Videokonferenz auf. Andere Behandler sind nur in Notfällen erreichbar, sodass in der Patientenversorgung erhebliche Defizite entstehen können.

Die Therapie mit Psychopharmaka sollte, v. a. zu Beginn der Behandlung, engmaschig ärztlich begleitet und kontrolliert werden. Sowohl die Evaluation der ausreichenden Wirksamkeit als auch die Beurteilung unerwünschter Arzneimittelwirkungen (UAW) sollten regelmäßig erfolgen. Selbst bei Patienten, die seit Jahren stabil auf eine Psychopharmakotherapie eingestellt sind, ist das Risiko einer Exazerbation einer vorbestehenden psychischen Erkrankung im Rahmen der COVID-19-Pandemie erhöht [28], was eine medikamentöse Optimierung erforderlich machen kann. Worauf sollte bezüglich der Psychopharmakotherapie derzeit geachtet werden, um sowohl eine suffiziente Behandlung als auch eine adäquate Arzneimitteltherapiesicherheit zu gewährleisten?

## Atemdepression und Pneumonie

Benzodiazepine stellen bei vielen psychischen Erkrankungen eine wirkungsvolle Option in der Akutbehandlung dar. Gerade bei Angsterkrankungen, deren Inzidenz ersten Studien zufolge im Rahmen der COVID-19-Pandemie zunimmt [28], wirken Benzodiazepine anxiolytisch, sodass Patienten rasch eine Symptomlinderung erfahren. Vor einer ärztlichen Verordnung von Benzodiazepinen sollten Patienten über die mögliche atemdepressive Wirkung aufgeklärt werden. Besteht eine Komedikation (z. B. mit Opioiden oder Antipsychotika), so sollte überprüft werden, inwiefern additive atemdepressive Effekte zu erwarten sind [10]. Hier kann eine Überprüfung möglicher Interaktionen mithilfe einer elektronischen Interaktionsdatenbank (z. B. AiD Klinik® [Dosing GmbH, Heidelberg, Deutschland], PSIAC [Springer-Verlag GmbH, Heidelberg, Deutschland] oder mediQ [Psychiatrische Dienste Aargau AG, Brugg, Schweiz]) hilfreich sein. Mit besonderer Vorsicht sollte die Anwendung potenziell atemdepressiver Substanzen bei älteren Patienten und Patienten mit pulmonalen Vorerkrankungen abgewogen werden. Ist keine geeignete Alternative verfügbar, so sollte die niedrigst wirksame Dosis über den kürzest möglichen Zeitraum gewählt werden, da bei der atemdepressiven Wirkung von einem dosis- bzw. konzentrationsabhängigen Effekt auszugehen ist [4]. Kurzwirksame Benzodiazepine ohne aktive Metabolite wie Lorazepam oder Oxazepam sollten bevorzugt werden, um das Kumulationsrisiko zu vermindern ([26]; Tab. 1).

**Table 1:** 

Wirkstoffgruppe	Substanzen (Bsp.)	UAW	Procedere und Risikoreduktion	Alternative Therapiemöglichkeiten
Benzodiazepine	Lorazepam Diazepam	Atemdepression, v. a. bei gemeinsamer Anwendung mit Clozapin, Opioiden etc.	Indikation überprüfen Niedrigst wirksame Dosis über den kürzest möglichen Zeitraum verwenden	Bevorzugte Verwendung kurzwirksamer Benzodiazepine (z. B. Lorazepam, Oxazepam) Niederpotente konventionelle Antipsychotika (z. B. Pipamperon, Melperon^a^)
Antipsychotika	Clozapin Olanzapin Quetiapin Risperidon Aripiprazol	Agranulozytose	Indikation überprüfen Komedikation auf hämatotoxische Substanzen prüfen (CAVE: Metamizol) Auf SARS-CoV-2-Infektion bei Mitpatienten achten Bei Neueinstellung von Clozapin: 3 × pro Woche Kontrolle des Differenzialblutbilds (vgl. Abb. 1)	Im Fall von Blutbildveränderungen Wahl eines anderen Antipsychotikums mit geringerem Risiko für Agranulozytose
	Clozapin Risperidon Quetiapin Pipamperon	Venöse Thromboembolien	Aufklärung der Patienten Abfragen möglicher Begleitsymptome und Risikofaktoren Ausreichende Flüssigkeitszufuhr Tragen von Kompressionsstrümpfen Aktivierung der Muskelpumpe Ggf. parenterale Antikoagulation	Absetzen nicht (mehr) indizierter Antipsychotika Ausweichen auf Antipsychotika, die mit einem niedrigeren Risiko für Thromboembolien assoziiert sind (z. B. Aripiprazol, Fluphenazin, Ziprasidon)
	Clozapin Risperidon	Pneumonien	Aufklärung der Patienten Bei Fieber oder grippalen Symptomen: Halbierung der Clozapin-Dosis Bei bestätigter SARS-CoV-2-Infektion: Reduktion der Clozapin-Dosis auf ein Drittel bzw. Absetzen erwägen	Ausweichen auf Antipsychotika, die mit einem niedrigeren Risiko für Pneumonien assoziiert sind (z. B. Aripiprazol, Quetiapin)
Antipsychotika Antidepressiva Antikonvulsiva	Clozapin Olanzapin Quetiapin Haloperidol Carbamazepin	Intoxikation durch interleukinvermittelte Hemmung der hepatischen Metabolisierung	Aufklärung der Patienten Abfragen möglicher Symptome (Infektion? UAW?) Kontrolle des Wirkstoffspiegels bei Infektion bzw. bei verändertem Rauchverhalten Dosisreduktion	Ggf. Ausweichen auf Präparate ohne relevante Metabolisierung über CYP3A4/CYP1A2
	Clozapin Olanzapin Mirtazapin Duloxetin	Intoxikation durch Reduktion des Tabakkonsums (Deinduktion der hepatischen Metabolisierung)		Bei Rauchern: falls möglich, Einsatz von Substanzen ohne relevante Metabolisierung über CYP1A2

*Bsp.* Beispiele, *CYP* Cytochrom-P450-Enzym, *SARS-CoV 2* severe acute respiratory syndrome coronavirus 2, *UAW* unerwünschte Arzneimittelwirkung

^a^Melperon ist ein Inhibitor von CYP2D6, sodass bei Komedikation mit CYP2D6-Substraten (z. B. Metoprolol) mit Wirkstoffspiegelerhöhungen zu rechnen ist

Bisher liegen keine Studien vor, die die Auswirkung von Benzodiazepinen im Rahmen akuter SARS-CoV-2-Infektionen untersucht haben, aus der verfügbaren Datenlage zur Anwendung von Benzodiazepinen bei Influenza oder grippalen Infekten ist jedoch bekannt, dass mit einem erhöhten Risiko für die Entwicklung einer Pneumonie, gegebenenfalls sogar mit einer erhöhten Mortalität zu rechnen ist [24]. Derartige Implikationen erscheinen pathomechanistisch plausibel und auf die Infektion mit SARS-CoV‑2 übertragbar.

Pneumonien und Infekte der oberen Atemwege werden bspw. in der Fachinformation von Risperidon und Clozapin als UAW aufgeführt [9]. Pneumonien zählen zu den häufigsten Todesursachen bei Patienten, die mit Clozapin behandelt werden, und weisen eine vierfach höhere Letalität auf als Clozapin-induzierte Agranulozytosen. Zwar liegen noch keine Berichte zur Behandlung mit Clozapin bei Patienten mit akuter SARS-CoV-2-Infektion vor, es wird jedoch vermutet, dass Clozapin über immunologische Mechanismen das Risiko für die Entwicklung einer Pneumonie erhöhen kann. Zudem führen die bei einer Infektion freiwerdenden Zytokine über eine Hemmung des Cytochrom-P450-Isoenzyms (CYP) 1A2 zu einer Erhöhung des Clozapin-Spiegels (für Details siehe Abschnitt „Veränderungen des Arzneistoffmetabolismus“). Hieraus resultiert eine bidirektionale Assoziation: Zum einen ist unter Clozapin das Risiko einer Pneumonie erhöht. Tritt unter Clozapin eine Pneumonie auf, erhöht sich andererseits das Risiko einer Clozapin-Intoxikation [18]. Daher sollten mit Clozapin behandelte Patienten und ggf. deren Angehörige darüber aufgeklärt bzw. daran erinnert werden, beim Auftreten von Fieber oder grippalen Symptomen unverzüglich den behandelnden Arzt zu kontaktieren [9]. Die Clozapin-Dosis sollte im Fall von Fieber/grippalen Symptomen halbiert werden. Bestätigt sich der Verdacht einer SARS-CoV-2-Pneumonie, so sollte kritisch die weitere Reduktion auf ein Drittel der Ausgangsdosis oder ein vollständiges Absetzen von Clozapin diskutiert werden [18].

Auch der Einsatz von Antipsychotika ist hinsichtlich einer atemdepressiven Wirkung kritisch zu überprüfen. Dieser Effekt kann bereits bei einer antipsychotischen Monotherapie eintreten, v. a. bei sedierenden Substanzen wie Clozapin und Olanzapin. Bei einer Kombinationstherapie mit Benzodiazepinen kann eine additive atemdepressive Wirkung resultieren, weshalb die Vorgaben der Fachinformation einzuhalten sind [9].

## Hämatotoxizität

Im Rahmen der COVID-19-Pandemie liegt ein besonderer Fokus auf der hämatotoxischen Wirkung von Psychopharmaka, welche meistens die neutrophilen Granulozyten (nG) betrifft. Der Schweregrad kann variabel sein, von einer milden Neutropenie (nG 1000–1499/µl) bis hin zu einer Agranulozytose (nG < 500/µl) [23], die mit einer erhöhten Infektanfälligkeit einhergeht [14]. Agranulozytose ist eine gefürchtete UAW von Clozapin, kann aber auch unter Therapie mit Quetiapin, Risperidon, Olanzapin, Aripiprazol, Perazin, Mirtazapin, Carbamazepin und Lamotrigin beobachtet werden. Die gemeinsame Anwendung der genannten Substanzen mit anderen potenziell hämatotoxischen Wirkstoffen, z. B. die Kombination von Clozapin und Valproat, kann das Risiko für eine Neutropenie erhöhen [9]. Metamizol findet aufgrund seiner potenten antipyretischen Wirkung u. a. bei fieberhaften Infektionen Anwendung [13]. Potenziell additive hämatotoxische Effekte von Psychopharmaka und Metamizol sollten berücksichtigt werden.

Die Inzidenz Clozapin-induzierter Agranulozytosen ist am höchsten in den ersten 6 Monaten nach Therapiebeginn [2], dennoch müssen die empfohlenen Blutbildkontrollen bei auf Clozapin eingestellten Patienten weiterhin monatlich erfolgen [9], v. a. wenn Patienten weitere hämatotoxische Substanzen einnehmen. In etwa 10 % der Fälle tritt eine Clozapin-induzierte Agranulozytose nach dem zweiten Behandlungsjahr auf [16]. Eine Verlängerung des Kontrollintervalls, z. B. auf 3 Monate [27], sollte kritisch überprüft werden. Die monatliche Durchführung von Blutbildkontrollen erscheint gegenwärtig unter Einhaltung der empfohlenen Hygienemaßnahmen umsetzbar.

**Figure 1:**
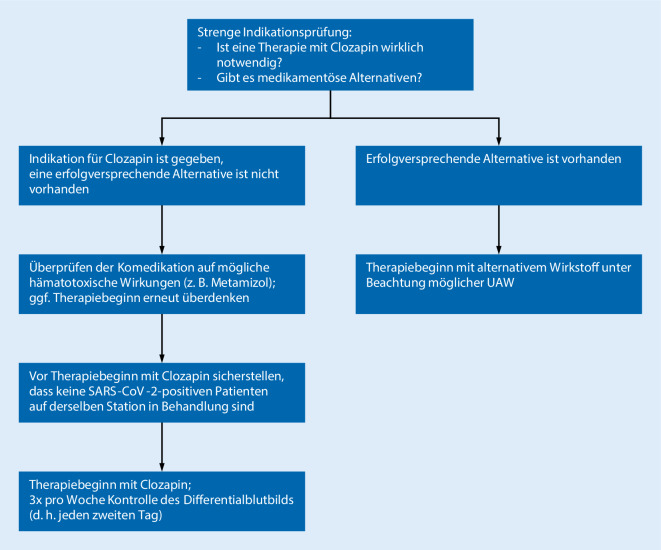

Die Neueinstellung eines Patienten auf Clozapin sollte aktuell nur erfolgen, wenn keine geeignete Behandlungsalternative besteht. Gleichzeitig sollte Clozapin Patienten mit therapieresistenter Schizophrenie nicht vorenthalten werden. Es sollten besondere Vorsichtsmaßnahmen bei der Therapieeinstellung berücksichtigt werden (Abb. 1).

## Thromboembolische Ereignisse

Eine akute Infektion kann das Risiko einer venösen Thromboembolie (VTE) vorübergehend erhöhen [7]. Hierzu tragen Immobilisation, Entzündungsprozesse, Hypoxie und diffuse intravaskuläre Koagulopathien bei. Unter intensivpflichtigen Patienten mit SARS-CoV-2-Infektion wurde eine hohe Inzidenz (31 %) an thromboembolischen Komplikationen beobachtet [15]. Die Behandlung mit Antipsychotika kann das Risiko für VTE v. a. bei Patienten, die älter als 65 Jahre sind, erhöhen [19], sodass im Fall einer Infektion mit SARS-CoV‑2 spezielle Präventionsmaßnahmen durchgeführt werden sollten ([25]; Tab. 1). Im Vergleich zu Patienten, die ein Antipsychotikum einnehmen, weisen Patienten, die mit ≥ 2 Antipsychotika behandelt werden, ein erhöhtes Risiko für VTE auf. Zudem sind Antipsychotika der zweiten Generation wie Risperidon, Clozapin und Quetiapin mit einem erhöhten Risiko für VTE assoziiert. Im Vergleich dazu treten VTE unter Aripiprazol, Ziprasidon und Fluphenazin seltener auf [19].

## Veränderungen des Arzneistoffmetabolismus

Bei einer Infektion mit Erhöhung des C‑reaktiven Proteins kann es zu Veränderungen des Arzneistoffmetabolismus über die Enzyme des CYP-Systems kommen. Die bei einer Infektion freigesetzten Zytokine können zu einer Hemmung des Abbaus von Psychopharmaka führen, sodass deren Wirkstoffkonzentrationen ansteigen [22]. Bei einer COVID-19-Infektion scheinen v. a. Interleukin(IL)-2 und IL‑6 von Bedeutung zu sein [21]. IL‑2 führt zu einer Reduktion der Expression von CYP1A2 und CYP3A4 [8], wohingegen IL‑6 die Expression von CYP3A4 hemmt [1]. Entsprechend sind Erhöhungen des Wirkstoffspiegels v. a. bei Wirkstoffen zu befürchten, die über CYP1A2 und CYP3A4 abgebaut werden, wie z. B. Olanzapin, Clozapin und Carbamazepin (Tab. 2; [26]). Es sollte bedacht werden, dass eine Hemmung der CYP-Enzyme mit geringer zeitlicher Latenz von teilweise nur einigen Stunden einsetzt und daher bereits wenige Tage nach Ausbruch einer Infektion mit Wirkstoffspiegelerhöhungen zu rechnen ist. Die zu erwartende Wirkstoffspiegelerhöhung kann 20–70 % betragen [1]. Bei Clozapin ist mit einer durchschnittlichen infektionsbedingten Erhöhung des Wirkstoffspiegels um 48 % zu rechnen, während bei Risperidon im Mittel ein 24-prozentiger Anstieg beobachtet wurde [12].

**Table 2:** 

Wirkstoff	Inhibition von CYP-Enzymen		Induktion von CYP-Enzymen
	CYP1A2	CYP3A4	CYP1A2
*Antipsychotika*			
Olanzapin	↑↑	–	↓↓
Clozapin	↑↑	↑	↓↓
Quetiapin	–	↑↑	–
Aripiprazol	–	↑↑	–
Risperidon	–	↑	–
Haloperidol	↑	↑↑	↓
Chlorpromazin	↑	↑	↓
*Antidepressiva*			
Duloxetin	↑↑	–	↓↓
Mirtazapin	↑↑	↑↑	↓↓
*Antikonvulsiva/Phasenprophylaktika*			
Carbamazepin	–	↑↑	–
*Antidementiva*			
Galantamin	–	↑	–
Donepezil	–	↑	–

Im Rahmen von Infektionen werden CYP1A2 und CYP3A4 durch IL‑2 [8], CYP3A4 zusätzlich durch IL‑6 inhibiert [1]. Tabakrauch ist ein potenter Induktor von CYP1A2 [3]. Dargestellt sind die zu erwartenden Wirkungen einer Inhibition/Induktion von CYP1A2 sowie einer Inhibition von CYP3A4 auf die Wirkstoffspiegel ausgewählter Psychopharmaka

↑ Anstieg des Wirkstoffspiegels zu erwarten

↑↑ Starker Anstieg des Wirkstoffspiegels zu erwarten

– Kein Einfluss auf den Wirkstoffspiegel zu erwarten

↓ Abfall des Wirkstoffspiegels zu erwarten

↓↓ Starker Abfall des Wirkstoffspiegels zu erwarten

*CYP* Cytochrom-P450-Enzym, *IL* Interleukin

Auch sollten die Auswirkungen einer möglichen Reduktion des Tabakkonsums während einer Infektion der Atemwege auf die Psychopharmakotherapie berücksichtigt werden. Die im Tabakrauch enthaltenen polyzyklischen aromatischen Kohlenwasserstoffe – nicht jedoch Nikotin – sind potente Induktoren von CYP1A2. Ein verringerter Tabakkonsum bzw. ein Rauchstopp kann eine Deinduktion von CYP1A2 bewirken und einen klinisch relevanten Anstieg des Wirkstoffspiegels von CYP1A2-Substraten nach sich ziehen [3]. Die Deinduktion von CYP1A2 tritt mit einer zeitlichen Latenz auf, sodass nach ca. einer Woche mit einer klinisch relevanten Erhöhung des Wirkstoffspiegels von CYP1A2-Substraten zu rechnen ist [3]. Ein Anstieg des Wirkstoffspiegels eines Pharmakons kann ein vermehrtes Auftreten von UAW sowie ggf. eine Medikamentenintoxikation bedingen. Auch stabil auf CYP1A2-Substrate eingestellte Patienten sollten hierauf aufmerksam gemacht werden, um – falls erforderlich – rechtzeitig eine Dosisanpassung einzuleiten (Tab. 1). E‑Zigaretten haben keine Auswirkung auf den Arzneistoffmetabolismus. Beim Umstieg von Zigarette (bzw. allgemein von Tabakprodukten) auf E‑Zigarette ist daher ebenfalls eine Deinduktion von CYP1A2 zu erwarten [3].

Im Rahmen einer SARS-CoV-2-Infektion können bakterielle Superinfektionen auftreten, die antibiotisch behandelt werden müssen [17]. Bei der Behandlung bakterieller Pneumonien kommen u. a. Makrolide wie Clarithromycin zum Einsatz, die aufgrund einer Hemmung von CYP3A4 zu einer Erhöhung des Wirkstoffspiegels von CYP3A4-Substraten wie Quetiapin führen können. Die gleiche Rationale gilt für die Medikamentenkombination Lopinavir/Ritonavir, welche in klinischen Studien an SARS-CoV-2-Infizierten getestet wurde [5]. Erfordert die klinische Symptomatik eines Patienten den Einsatz eines Medikaments mit CYP3A4-hemmender Eigenschaft wie Clarithromycin oder Lopinavir/Ritonavir, so sollte Quetiapin pausiert werden. Clozapin, wenn auch in deutlich geringerem Ausmaß, und andere über CYP3A4 ganz oder teilweise metabolisierte Substanzen sind von dieser pharmakokinetischen Interaktion betroffen. Neben Clarithromycin wird auch das Fluorchinolon Ciprofloxacin, ein Inhibitor von CYP1A2 und CYP3A4, bei bakteriellen (Super‑)Infektionen der Atemwege eingesetzt. Eine Komedikation mit Substraten dieser CYP-Enzyme sollte unter Beachtung möglicher Wirkstoffspiegelerhöhungen erfolgen ([26]; Tab. 2).

## Neueinstellung auf Psychopharmaka

Erste Daten aus China zeigen, dass die COVID-19-Pandemie und die damit einhergehenden Veränderungen in nahezu allen Lebensbereichen eine Zunahme psychischer Symptome bedingt [28]. Aufgrund der eingeschränkten Infrastruktur der Krankenversorgung sind zahlreiche nichtmedikamentöse Behandlungsoptionen derzeit nur eingeschränkt verfügbar. Hierzu zählen ambulante und stationäre Patientengruppen, Arbeits- und Ergotherapie sowie Selbsthilfegruppen. Darüber hinaus ist die Suche nach einer ambulanten Psychotherapie derzeit erschwert. So kann es in Ermangelung alternativer Angebote notwendig sein, eine Psychopharmakotherapie zu etablieren oder zu intensivieren. Hierbei sind eine sorgfältige Risiko-Nutzen-Abwägung sowie eine ausführliche Aufklärung über mögliche UAW von besonderer Bedeutung. Viele Arztkontakte finden aktuell nur telefonisch oder per Videokonferenz statt, sodass die klinische Untersuchung des Patienten mit Fokus auf potenzielle UAW erschwert ist. Patienten sollten explizit nach Symptomen befragt werden, die verdächtig auf UAW sind. Darüber hinaus sind die erforderlichen Vor- und Kontrolluntersuchungen (u. a. EKG, EEG, Laboruntersuchungen) weiterhin durchzuführen.

In Anbetracht des charakteristischen Symptoms eines veränderten Geruchs- bzw. Geschmackssinns bei COVID-19 [29] sollte bei einer Neueinstellung von Patienten auf Lithium, Asenapin und Zopiclon darauf hingewiesen werden, dass es zu Geschmacksveränderungen kommen kann [9].

## Remdesivir

Remdesivir ist ein Hemmstoff viraler RNA-Polymerasen, der gegenwärtig in klinischen Studien an SARS-CoV-2-Infizierten getestet wird [11]. Bisher wurde noch nicht über pharmakokinetische Arzneimittelinteraktionen von Remdesivir berichtet. Pharmakodynamische Wechselwirkungen von Remdesivir mit Psychopharmaka sind vorstellbar. So traten bei 4 von 53 der in einer klinischen Studie mit Remdesivir behandelten Patienten (8 %) Hypotonien, bei 6 % tiefe Venenthrombosen (TVT) und bei 4 % Delirien auf [11]. Hieraus ergeben sich potenzielle Arzneimittelinteraktionen u. a. mit Antipsychotika (erhöhtes Risiko für Hypotonien, TVT und Delirien), trizyklischen Antidepressiva (Hypotonien und Delirien) und Benzodiazepinen (Delirien; [26]).

## (Hydroxy‑)Chloroquin

Die Malariamittel Chloroquin und Hydroxychloroquin haben einen festen Stellenwert in der Rheumatologie [9] und werden aufgrund potenziell antiviraler Effekte im Rahmen klinischer Studien zur Behandlung von COVID-19 getestet [6]. (Hydroxy‑)Chloroquin kann v. a. bei älteren Menschen neuropsychiatrische UAW bis hin zu Psychosen auslösen [20]. Darüber hinaus kann (Hydroxy‑)Chloroquin die Anfallsschwelle senken, die QTc-Zeit verlängern und CYP2D6 inhibieren [9], worüber vielfältige pharmakodynamische und -kinetische Interaktionen mit Psychopharmaka möglich sind. Aufgrund der großen Medienpräsenz von (Hydroxy‑)Chloroquin in den vergangenen Wochen ist es denkbar, dass Menschen aus Sorge vor einer Ansteckung mit SARS-CoV‑2 selbstständig eine Medikation mit (Hydroxy‑)Chloroquin einleiten oder Patienten mit rheumatologischer Erkrankung ihre bereits bestehende Therapie mit (Hydroxy‑)Chloroquin steigern. In beiden Szenarien könnte es unbeabsichtigt zum Auftreten psychiatrischer UAW kommen. Diese Überlegungen veranschaulichen, dass eine detaillierte Medikamentenanamnese essenziell in der Abklärung neu aufgetretener psychiatrischer Symptome ist. (Hydroxy‑)Chloroquin sollte aus den genannten Gründen nur bei gegebener (rheumatologischer) Indikation in der ärztlich verordneten Dosierung oder innerhalb klinischer Studien zu COVID-19 verabreicht werden.

## Fazit für die Praxis

- Wirkstoffe mit atemdepressiver Wirkung (z. B. Benzodiazepine) sollten bei älteren und pulmonal vorerkrankten Patienten zurückhaltend eingesetzt werden.
- Pneumonien zählen zu den häufigsten Todesursachen bei mit Clozapin behandelten Patienten.
- Zahlreiche Psychopharmaka können hämatotoxische Effekte ausüben. Insbesondere die Neueinstellung auf Clozapin sollte vor dem Hintergrund des bekannten Agranulozytoserisikos sorgfältig abgewogen werden.
- Die Behandlung mit Antipsychotika kann das Risiko für venöse Thromboembolien erhöhen.
- Durch eine Infektion kann es zu einer Hemmung des Arzneistoffmetabolismus kommen, sodass mit erhöhten Wirkstoffspiegeln bis hin zu Intoxikationen zu rechnen ist. Dosisreduktionen sind bei Medikamenten wie Clozapin beim Auftreten von Fieber bereits vor Erhalt des Medikamentenspiegels vorzunehmen.
- Die Reduktion des Tabakkonsums bei einer Infektion der Atemwege kann zu einem Anstieg der Wirkstoffspiegel von Cytochrom-P450-Isoenzym(CYP)1A2-Substraten führen.
- Im Rahmen von (telefonischen) Patientenkontakten sollte aktiv nach unerwünschten Arzneimittelwirkungen gefragt werden.

## References

[1] Aitken AE, Richardson TA, Morgan ET (2006). Regulation of drug-metabolizing enzymes and transporters in inflammation. Annu Rev Pharmacol Toxicol.

[2] Alvir JM, Lieberman JA, Safferman AZ (1993). Clozapine-induced agranulocytosis. Incidence and risk factors in the United States. N Engl J Med.

[3] Anderson GD, Chan LN (2016). Pharmacokinetic drug interactions with tobacco, cannabinoids and smoking cessation products. Clin Pharmacokinet.

[4] Battaglia S, Bezzi M, Sferrazza Papa GF (2014). Are benzodiazepines and opioids really safe in patients with severe COPD?. Minerva Med.

[5] Cao B, Wang Y, Wen D (2020). A trial of lopinavir-ritonavir in adults hospitalized with severe Covid-19. N Engl J Med.

[6] Cortegiani A, Ingoglia G, Ippolito M (2020). A systematic review on the efficacy and safety of chloroquine for the treatment of COVID-19. J Crit Care.

[7] Danzi GB, Loffi M, Galeazzi G (2020). Acute pulmonary embolism and COVID-19 pneumonia: a random association?. Eur Heart J.

[8] Elkahwaji J, Robin MA, Berson A (1999). Decrease in hepatic cytochrome P450 after interleukin-2 immunotherapy. Biochem Pharmacol.

[9] Fachinfo-Service Fachinformationsverzeichnis Deutschland (2020) Webpräsenz. www.fachinfo.de. Zugegriffen: 21. Apr. 2020

[10] Golčić M, Dobrila-Dintinjana R, Golčić G (2018). The impact of combined use of opioids, antipsychotics, and anxiolytics on survival in the hospice setting. J Pain Symptom Manage.

[11] Grein J, Ohmagari N, Shin D (2020). Compassionate use of remdesivir for patients with severe Covid-19. N Engl J Med.

[12] Hefner G, Shams ME, Unterecker S (2016). Inflammation and psychotropic drugs: the relationship between C-reactive protein and antipsychotic drug levels. Psychopharmacol (Berl).

[13] Huber M, Andersohn F, Sarganas G (2015). Metamizole-induced agranulocytosis revisited: results from the prospective Berlin case-control surveillance study. Eur J Clin Pharmacol.

[14] Klastersky JA, Meert AP (2016). Understanding the risk for infection in patients with neutropenia. Intensive Care Med.

[15] Klok FA, Kruip M, van der Meer NJM (2020). Incidence of thrombotic complications in critically ill ICU patients with COVID-19. Thromb Res.

[16] Lahdelma L, Appelberg B (2012). Clozapine-induced agranulocytosis in Finland, 1982–2007: long-term monitoring of patients is still warranted. J Clin Psychiatry.

[17] Lake MA (2020). What we know so far: COVID-19 current clinical knowledge and research. Clin Med (Lond).

[18] de Leon J, Ruan CJ, Schoretsanitis G (2020). A rational use of clozapine based on adverse drug reactions, pharmacokinetics, and clinical pharmacopsychology. Psychother Psychosom.

[19] Letmaier M, Grohmann R, Kren C (2018). Venous thromboembolism during treatment with antipsychotics: results of a drug surveillance programme. World J Biol Psychiatry.

[20] Mascolo A, Berrino PM, Gareri P (2018). Neuropsychiatric clinical manifestations in elderly patients treated with hydroxychloroquine: a review article. Inflammopharmacol.

[21] McGonagle D, Sharif K, O’Regan A (2020). The role of cytokines including Interleukin-6 in COVID-19 induced pneumonia and macrophage activation syndrome-like disease. Autoimmun Rev.

[22] Morgan ET (2009). Impact of infectious and inflammatory disease on cytochrome P450-mediated drug metabolism and pharmacokinetics. Clin Pharmacol Ther.

[23] Myles N, Myles H, Xia S (2018). Meta-analysis examining the epidemiology of clozapine-associated neutropenia. Acta Psychiatr Scand.

[24] Nakafero G, Sanders RD, Nguyen-Van-Tam JS (2016). The association between benzodiazepines and influenza-like illness-related pneumonia and mortality: a survival analysis using UK primary care data. Pharmacoepidemiol Drug Saf.

[25] Ogłodek EA, Just MJ, Grzesińska AD (2018). The impact of antipsychotics as a risk factor for thromboembolism. Pharmacol Rep.

[26] Procyshyn RM, Bezchilbynk-Butler KZ, Jeffries JJ (2019). Clinical handbook of psychotropic drugs.

[27] Siskind D, Honer WG, Clark S (2020). Consensus statement on the use of clozapine during the COVID-19 pandemic. J Psychiatry Neurosci.

[28] Wang C, Pan R, Wan X (2020). Immediate psychological responses and associated factors during the initial stage of the 2019 coronavirus disease (COVID-19) epidemic among the general population in China. Int J Environ Res Public Health.

[29] Yan CH, Faraji F, Prajapati DP (2020). Association of chemosensory dysfunction and Covid-19 in patients presenting with influenza-like symptoms. Int Forum Allergy Rhinol.

